# *Mycobacterium tuberculosis* Infects Human Visceral White Adipocytes and Expresses Dormancy Genes and Inflammatory Cytokines: The Role of Visceral Adipocytes in Latent Tuberculosis Infection

**DOI:** 10.3390/ijms262311595

**Published:** 2025-11-29

**Authors:** Ana E. Garduño-Torres, Manuel G. Salgado-Cantú, Silvia Guzmán-Beltrán, Jesús Montoya-Ramírez, Juan Antonio Suárez-Cuenca, Enrique Ortega, David Ricardo Orozco-Solís, Daniela I. Uribe-López, María Teresa Herrera, Luis Horacio Gutiérrez-González, Yolanda González

**Affiliations:** 1 Department of Microbiology, Instituto Nacional de Enfermedades Respiratorias Ismael Cosío Villegas, Mexico City 14080, Mexico; anagt@ciencias.unam.mx (A.E.G.-T.);; 2 Bariatric Surgery Clinic, Centro Médico Nacional “20 de Noviembre”, Instituto de Seguridad y Servicios Sociales de los Trabajadores del Estado, Mexico City 03104, Mexico; 3 Laboratory of Experimental Metabolism and Clinical Research, Department of Clinical Research, Centro Médico Nacional “20 de Noviembre”, Instituto de Seguridad y Servicios Sociales de los Trabajadores del Estado, Mexico City 03104, Mexico; 4 Department of Immunology, Instituto de Investigaciones Biomédicas, Universidad Nacional Autónoma de México, Mexico City 04510, Mexico; 5 Laboratorio de Cronobiología, Metabolismo y Envejecimiento, Instituto Nacional de Medicina Genómica, Centro de Investigación sobre el Envejecimiento-Centro de Investigación y de Estudios Avanzados del Instituto Politécnico Nacional, Mexico City 14330, Mexico; 6 Laboratory of Transcriptomics and Molecular Immunology, Instituto Nacional de Enfermedades Respiratorias Ismael Cosío Villegas, Mexico City 14080, Mexico; lhgut@iner.gob.mx

**Keywords:** latent tuberculosis infection, *Mycobacterium tuberculosis*, human visceral white adipocytes, dormancy-associate genes

## Abstract

This study explores the role of human visceral white adipocytes (hv-WAD) in latent tuberculosis infection (LTBI). While granulomas and macrophages are traditionally viewed as central to TB latency, emerging evidence highlights adipocytes as significant non-canonical host cells that may facilitate bacterial persistence by providing a protective niche. Unlike the immune-driven environment within granulomas, adipocytes can shield *Mycobacterium tuberculosis* (Mtb) from immune surveillance, promoting survival. In vitro experiments showed that Mtb invades approximately 39% of hv-WAD within 48–72 h post-infection (hpi). Both avirulent H37Ra and virulent H37Rv Mtb strains, when infecting adipocytes, expressed RNA for key virulence factors (19 kDa, 30 kDa, Ag85b, 5KST, CFP10, and ESAT6) and dormancy-associated genes (Icl1, LipY, WhiB3, SodA, and Tgs1) at 72 hpi. Infection stimulated the production of inflammatory cytokines, notably leading to a fivefold increase in TNF-α with H37Rv (*p* < 0.01). Additionally, we detected Mtb RNA transcripts (IS6110, 5KST, 30 kDa, CFP10, Ag85) in 68% of biopsies from TB asymptomatic patients. The transcripts suggest a metabolically heterogeneous state of mycobacteria. These findings position visceral fat as a potential reservoir for Mtb in latent TB infection and underscore the development of novel diagnostic strategies targeting adipose tissue.

## 1. Introduction

Latent tuberculosis infection (LTBI) remains a global health challenge. It is estimated that approximately 25% of the world’s population harbors LTBI. Recent research has reported that, up to 12 years after initial contact with an infected individual, household members may still be at risk of developing active tuberculosis (TB) [1,2,3]. LTBI is defined as a state in which there is a persistent immune response to *Mycobacterium tuberculosis* (Mtb) antigens, with no clinical or radiological evidence of active disease. During this latent phase, Mtb can potentially reactivate, especially in individuals with compromised immune systems, leading to active TB. Moreover, Mtb employs multiple mechanisms to evade the immune system and cause active disease in susceptible individuals [4]. This ability to persist without causing active disease makes LTBI a critical target for TB control efforts worldwide [5].

For decades, granulomas (organized aggregates of immune cells) have been recognized as key structures that maintain Mtb in a long-term dormant state. Alveolar macrophages play an important role in granuloma formation (in the pulmonary parenchyma, the establishment of infected macrophages triggers a chronic inflammatory response; this process involves the recruitment of uninfected macrophages, culminating in the organization of the granuloma), where mycobacteria enter a non-replicating, persistent state under immune control [6]. However, when this tightly regulated host–pathogen balance is disrupted, bacterial reactivation can occur, leading to the release of viable bacteria and the development of active TB [5]. Recent evidence reveals that Mtb can also infect non-hematopoietic cells, such as adipocytes [7]. These cells possess limited antimicrobial defenses compared to professional immune cells, including reduced production of reactive oxygen species, antimicrobial peptides, and impaired phagolysosomal maturation. This permissive environment likely enables Mtb to evade immune surveillance, thereby establishing a protected niche that facilitates long-term bacterial persistence [8,9,10,11].

To ensure intracellular survival, Mtb adopts a dormant state characterized by reduced metabolic activity. During this phase, Mtb minimizes protein synthesis while preserving essential cellular functions such as cell wall integrity, membrane potential, and genome stability, all while evading host immune detection. For nutrient acquisition, Mtb primarily relies on fatty acids, particularly triglycerides and cholesterol, as energy sources. In macrophages, Mtb induces the accumulation of cytoplasmic lipid droplets, which form lipid-rich structures reminiscent of adipose tissue. Additionally, both in vitro and in vivo studies, utilizing 3T3-L1 murine cells or white pre-adipose tissue (WAT) from animal models, have demonstrated that Mtb can infect these lipid-rich cells [8,12,13].

Unlike macrophages, which need to activate lipid catabolism genes to mobilize stored lipids, adipocytes are specialized for fat storage and offer a nutrient-rich environment that promotes Mtb survival and persistence by supplying abundant lipids for bacterial metabolism [8,9,12,14,15,16].

A key survival strategy that enables Mtb long-term persistence within the host is its remarkable ability to enter dormancy. During granuloma formation, the pathogen significantly upregulates two essential lipid metabolic pathways: lipid biosynthesis including genes such as Tgs-1 and Ppe4, a perilipin-like protein involved in triacylglycerol synthesis and lipid hydrolysis, mediated by enzymes like LipY, Msh1, and multiple lipases such as lip -F, -H, -J, -K, -N, -V, and -X, as well as culp 5–7 lipases/esterase [14,16,17,18]. In both human Simpson–Golabi–Behmel syndrome (SGBS) preadipocytes and the 3T3-L1 murine cell line differentiated into adipose cells, Mtb infection induces the expression of dormancy-associated genes such as dosR and hspX within 48 h post-infection (hpi) [19]. This coordinated genetic reprogramming allows Mtb to adapt and survive in the nutrient-deprived, intracellular environment.

However, dormancy is not a passive state of bacterial inactivity; it is a complex, active process of adaptation that enables Mtb to survive for extended periods within a functioning host-immune system. Infection of 3T3-L1-derived adipocytes with Mtb has been shown to induce the secretion of cytokines such as IL-6 and TNF-α at low bacterial concentrations [7]. Furthermore, a rabbit model demonstrated that Mtb infection increased the mRNA levels of TNF-α, IL-6, and IL-10 in white adipose tissue (WAT) [13]. These findings suggest that the combined actions of metabolic adaptation and immune evasion are essential for establishing and maintaining Mtb dormancy within adipocytes.

This study investigates Mtb infection in primary human visceral white adipocytes (hv-WAD) from biopsies of bariatric surgery patients, using both the attenuated H37Ra strain and the virulent strain H37Rv. We observed that 39% of hv-WAD were infected within 48 hpi. Transcriptional analysis suggests that the detected transcripts likely reflect a metabolically heterogeneous Mtb population within infected adipocytes. In this model, distinct bacterial subsets express specialized gene programs encompassing virulence factors and dormancy-associated genes, along with the secretion of pro-inflammatory cytokines TNF-α and IL-1β. Therapeutic agents that inhibit TNF-α, a key cytokine in the host response to infection, are associated with the reactivation of latent tuberculosis [20]. These findings demonstrate that adipocytes alone can support Mtb persistence, establishing a proinflammatory niche capable of maintaining bacterial dormancy in the absence of other immune cell types, and might contribute to latent TB establishment or markers for latent infection diagnosis.

## 2. Results

### 2.1. Mtb Infects hv-WAD Cells

**Figure 1:**
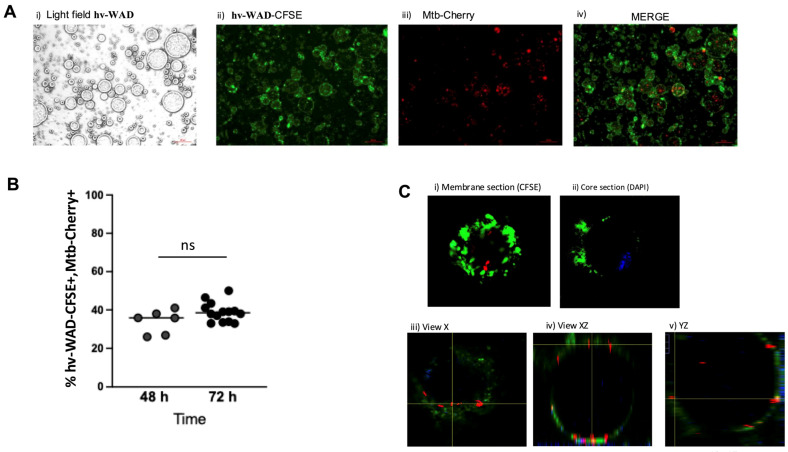
Mtb is internalized into hv-WAD. (**A**) Light field image of hv-WAD (i), hv-WAD stained with CFSE (ii), hv-WAD infected with Mtb-H37Ra-Cherry (MOI 20, 72 h) (iii), and merged image of *Mtb* with Cherry and hv-WAD-CFSE (iv), captured by fluorescence microscopy. (**B**) Infection percentage of hv-WADs infected with Mtb H37Ra-Cherry at 48 h (n = 6) and 72 h (n = 14). The dashed horizontal line indicates the median, and the Mann–Whitney test was used for statistical comparison, ns = non-significant (**C**) Orthogonal confocal views of hv-WAD stained with CFSE (membrane/cytoplasm) (i) and DAPI (nucleus) (ii), infected with Mtb-H37Ra-Cherry (red). Views include X (iii), XZ (iv), and YZ (v), visualized under a confocal microscope.

We first evaluated Mtb H37Ra-Cherry internalization in hv-WAD by microscopy. Results are shown in Figure 1. The first picture in panel A shows individual hv-WAD under light microscopy. To distinguish live hv-WAD from free lipid droplets, membrane-CFSE staining was used to identify the cells (panel A, second image). Bacteria were visualized by m-Cherry fluorescence (C). Combining these fluorescence images, we were able to calculate the percentage of hv-WAD containing Mtb. An average of 500–750 cells were counted per sample. At 48 hpi, 29.75% (range: 4.55–41.36) of CFSE-positive hv-WAD were Mtb H37Ra-Cherry positive. This percentage increased to 38.54% (range: 27.5–50) at 72 hpi, with no significant further increase in bacterial uptake (B). To confirm intracellular localization and exclude surface adhesion, different tissue sections were analyzed: membrane, core, and views in X, XZ, and XY planes. The images showed Mtb colocalizing near the nucleus and inside the hv-WAD (C).

### 2.2. RNA Transcripts Expressed in hv-WAD

**Figure 2:**
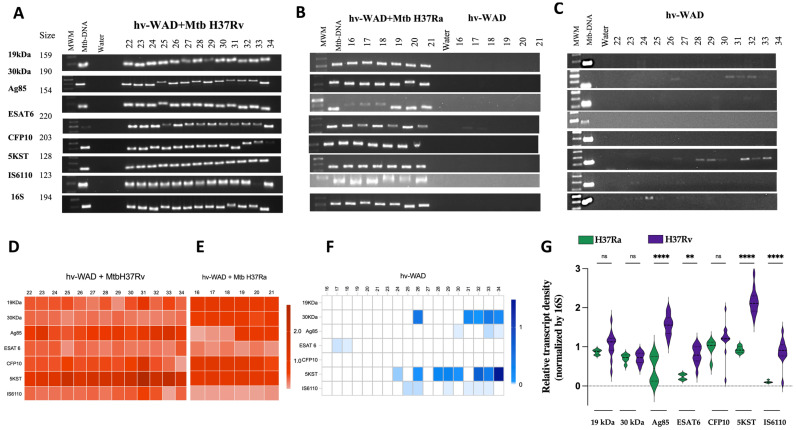
hv-WAD contains RNA sequences of Mtb antigens. RT-PCR experiments from hv-WAD showing the expression of Mtb genes: 19 kDa, 30 kDa, Ag85, ESAT6, CFP10, 5KST, IS6110 or 16S rRNA. Panels A-C present the PCR results under different conditions: (**A**) hv-WAD infected with the virulent Mtb-H37Rv strain (n = 13). (**B**) hv-WAD infected with the attenuated Mtb-H37Ra strain (left) (n = 6) or non-infected hv-WAD (right) (n = 6). (**C**) hv-WAD without infection (n = 13). Panels (**D**–**F**) correspond to densitometric intensity band normalized to 16S rRNA. (**G**) shows a violin plot of the relative transcript density for strains H37Ra versus H37Rv. Asterisks indicate a significance level of *p* ** *p* = 0.001, **** *p* < 0.0001, ns = non-significant as determined by ANOVA test. Molecular weight marker (MWM), Mtb-DNA (positive control) and water (negative control).

To further assess the functional activity of Mtb within adipocytes, we analyzed bacterial gene expression, we amplified Mtb RNA transcripts from infected hv-WAD using reverse transcription PCR (RT-PCR). hv-WADs were infected with both strains, H37Ra (n = 6) and H37Rv (n = 13), for 72 h. Infections with both strains resulted in the detection of RNA sequences for multiple antigens, including 19 kDa, 30 kDa, Ag85, ESAT6, CFP10, 5KST, IS6110 or 16S (Figure 2A,B). Despite varied gene amplification among patients, the results show that Mtb remains active within adipocytes (Figure 2D–F). To determine if the transcription of Mtb antigen-encoding RNAs differed between the H37Ra and H37Rv strains, we compared their amplification levels and found that Ag85, ESAT6, 5KST, and IS6110 were significantly more induced by the virulent H37Rv strain (Figure 2G).

**Figure 3:**
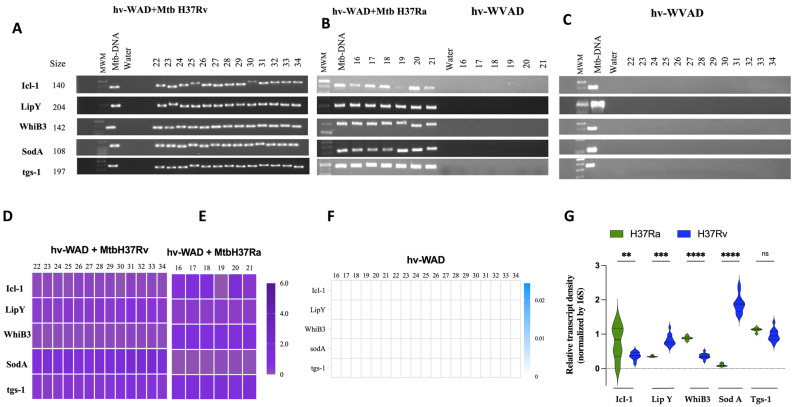
Mtb express dormancy-related genes within hv-WAD adipocytes. RNA was extracted from hv-WAD and reverse-transcribed into cDNA. RT-PCR experiments showing the expression of the Mtb genes: Icl-1, LipY, WhiB3, SodA and Tgs-1. Panels A-C present the PCR results under different conditions: (**A**) hv-WAD infected with the virulent Mtb-H37Rv strain (n = 13). (**B**) hv-WAD infected with the attenuated Mtb-H37Ra strain (left) or uninfected (right) (n = 6). (**C**) Uninfected hv-WAD control (n = 13). Panels (**D**–**F**) correspond to densitometric intensity band normalized to 16S rRNA. (**G**) shows a violin plot of the relative transcript density for strains H37Ra versus H37Rv. Asterisks indicate a significance level of *p* ** *p* = 0.001, *** *p* = 0.0001, **** *p* < 0.0001, ns = non-significant as determined by ANOVA test. Molecular weight marker (MWM), Mtb-DNA (positive control) and water (negative control).

Furthermore, we evaluated the presence of mRNA from dormancy-associated Mtb genes in hv-WAD infected with both strains. RNA for Icl-1, LipY, WhiB3, SodA and Tgs-1 was detected in all infected samples (Figure 3A,B). The relative expression levels varied among patients, as indicated by differences in gene amplification density (Figure 3D,E). To evaluate differences in the transcription of dormancy-associated Mtb antigen genes between the H37Ra and H37Rv strains, we compared their expression levels. We found that Icl1 and WhiB3 were significantly more induced in the attenuated H37Ra strain, whereas LypY and SodA were preferentially induced by the virulent H37Rv strain (Figure 3G).

Furthermore, Mtb RNA was detected in 68% of the adipose tissue biopsies, despite a lack of overt clinical disease. Specifically, patients 22 and 25, showed RNA for IS6110; patient 24 expressed RNA for 5KST; patients 26 and 32 had RNA for IS6110, 5KST, and the 30 kDa; patients 28 and 30 expressed RNA for 5KST; patient 29 showed RNA for IS6110 and 5KST; patient 30 expressed RNA for IS6110 and the 30 kDa; patient 33 had RNA for IS6110, Ag85 and 30 kDa, and 5KST; and patient 34 expressed RNA for Ag85 kDa, 19 kDa, and 30 kDa and patient 32 and 33, expressed RNA for ESAT-6 (Figure 2C,F). We also assessed transcripts from Mtb genes associated with dormancy, including Icl-1, LipY, WhiB3, SodA and Tgs-1. No expression of these dormancy-related genes was detected in the samples (Figure 3C,F). This finding supports the notion that Mtb is capable of remaining hidden within the adipose tissue of susceptible patients with metabolic imbalances.

### 2.3. Proinflammatory Cytokines in Cultures of hv-WAD

**Figure 4:**
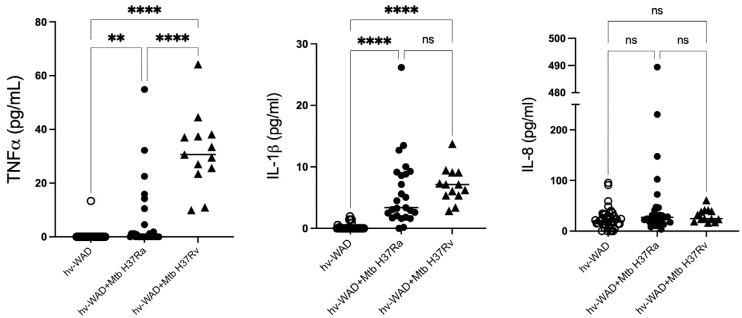
Quantification of inflammatory cytokines in Mtb-infected hv-WAD supernatants. hv-WAD isolated from patient biopsies were left uninfected (n = 54) or were infected with *M. tuberculosis* strains H37Rv (n = 13) or H37Ra (n = 41). After 72 h, the concentrations of the inflammatory cytokines TNF-α, IL-1β, and IL-8 were measured in the culture supernatants. Data are presented as scatter plots with medians. Statistical comparisons between groups were performed using the Wilcoxon signed-rank test in GraphPad Prism (v10.4.0, Boston, MA, USA). Asterisks indicate a significance level of *p*: ** *p* = 0.001, **** *p* < 0.0001, ns=not significant.

**Figure 5:**
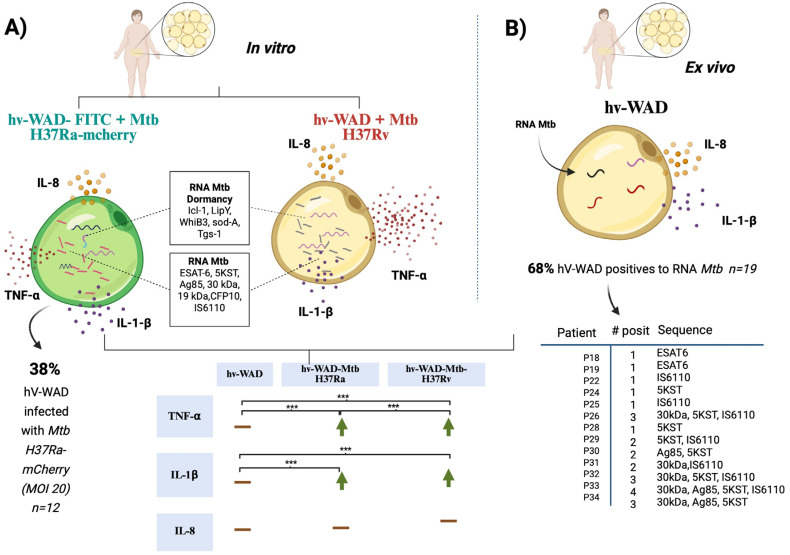
Mycobacterium tuberculosis Invasion of hv-WAD Cells. (**A**) In vitro infection model. Human visceral white adipocytes (hv-WAD) were infected with Mtb strains H37Ra-mCherry or H37Rv. Infection was confirmed by the detection of Mtb- RNAs, including: 19 kDa, 30 kDa, Ag85, ESAT6, CFP10, 5KST, IS6110, and dormancy-associated genes: Icl-1, LipY, WhiB3, SodA, Tgs-1. Infected hv-WADs also secreted elevated levels of the inflammatory cytokines TNF-α andIL-1β, compared to uninfected hV-WAD. (**B**) Ex vivo analysis of patient-derived hv-WADs. Uninfected hv-WADs, isolated from patient biopsies, express Mtb RNA sequences and constitutively secreted IL-8. Data are presented as scatter plots with medians. Statistical comparisons between groups were performed using the Wilcoxon signed-rank test in GraphPad Prism (v10.4.0, Boston, MA, USA); Asterisks indicate a significance level *p* < 0.05. Created in BioRender, https://app.biorender.com/; accessed on 17 September 2025.

The microenvironment, particularly inflammatory cytokines, is known to be essential for the maintenance of latent TB. In this study, we measured the secretion levels of IL-1β, IL-8, and TNF-α in media from hv-WAD infected with Mtb strains H37Ra or H37Rv. post-infection, hv-WAD significantly increased the production of TNF-α and IL-1β (*p* < 0.01) in response to both strains. Notably, H37Rv induced a fivefold higher increase in TNF-α compared to H37Ra (*p* < 0.01). Uninfected samples from patients exhibited constitutive IL-8 secretion, with low levels of IL-1β (Figure 4). The results are summarized in the Figure 5.

## 3. Discussion

Adipocyte-derived cell lines, such as SGBS, preadipocytes or the 3T3-L1 murine fibroblasts (differentiated into preadipocytes), have provided most of the current data on Mtb infection [7,8,13,19]. However, studies using primary cells from human patients remain scarce. In this study, we infected primary hv-WAD obtained from individuals undergoing bariatric surgery. We observed a maximum infection rate of 50% when infected with Mtb-H37Ra. In contrast, earlier experiments involving SGBS and 3T3-L1 cell lines reported infection rates of approximately 80% with MOI 20 and about 10% with MOI 1, respectively. Discrepancies in the results may be due to experimental variables, including the model organism (human versus mouse), the specific cell type used (preadipocytes versus fibroblast-preadipocytes), the bacterial strain and multiplicity of infection (MOI 20 versus MOI 1), and the incubation time (4 h versus 24 h). Additionally, the inherent differences between visceral and subcutaneous adipocytes may influence their susceptibility to Mtb infection [8,11,13]. Our findings demonstrate that Mtb can infect adipocytes, given their significantly longer lifespan (mean ~9.5 years), adipocytes could potentially serve as long-term reservoirs for the bacteria. However, both preadipocytes and mature adipocytes are susceptible to infection by Mtb.

When Mtb infects hv-WADs, it begins expressing RNA transcripts closely associated with dormancy. Hypoxia in intracellular bacteria has been shown to promote an increase in the expression of triacylglycerol synthase 1 (Tgs-1) and isocitrate lyase (Icl-1) just before the onset of dormancy, while lipase Y (LipY) expression rises during the mid-phase, and WhiB3 responds to environmental signals [21,22]. Additionally, superoxide dismutase helps protect the bacteria against various stress factors and is abundant during dormancy [23]. Our findings indicate that both Mtb H37Ra and H37Rv, when inside hv-WADs, induce the expression of RNA for Icl-1, LipY, WhiB3, SodA and Tgs-1. Comparison of transcriptional profiles between the H37Ra and H37Rv strains revealed distinct expression patterns. The virulent H37Rv strain significantly upregulated the transcripts Ag85, ESAT6, 5KST, IS6110, LipY, and SodA. In contrast, the attenuated H37Ra strain showed significantly higher expression of the dormancy-associated transcripts Icl1 and WhiB3. No differences in gene expression were observed between donors with and without type 2 diabetes or across different obesity classes. This suggests that these bacteria undergo metabolic reprogramming associated with the transition to a dormant state.

In addition to dormancy-related genes, Mtb also induces immunomodulatory antigens that facilitate immune evasion within adipose tissue. These include the 19 kDa lipoprotein and the 30 kDa component of the Ag85 complex (FbpB and FbpC), the ESAT-6/CFP-10 complex, and the 5Kbp sequence (5KST). The 5Kbp sequence specific to the *M. tuberculosis* complex is used diagnostically for tuberculosis, but it has no known functional role [24]. CFP-10 is especially significant because it forms a complex with ESAT-6, and this CFP-10/ESAT-6 complex’s interaction with cell surface receptors may modulate host cell behavior [25,26]. The Ag85 complex (comprising diacylglycerol acyltransferase/mycolyltransferase proteins such as FbpA, FbpB, and FbpC) is crucial for the synthesis of the mycobacterial cell wall. These proteins function as mycolyl-transferases, enzymes that are essential for synthesizing the waxy, lipid-rich outer layer of the cell wall. This thick cell wall is critical for Mtb’s virulence and its ability to resist host immune defenses and antibiotic treatment [24,27]. The diagnosis of LTBI relies on indirect tests that detect the immune response to Mtb antigens. The most common blood test is the Interferon-Gamma Release Assay (IGRA), which includes the CFP-10, ESAT-6, and TB7.7 (Rv2654) antigens [28,29]. These findings demonstrate that the immune system actively surveils Mtb antigens originating from adipocytes, a phenomenon that can be reproduced in vitro. However, the precise subcellular localization of these transcripts in adipocytes remains an important open question.

Furthermore, the maintenance of LTBI critically depends on a specific microenvironment. Studies have shown that administration of TNF-α inhibitors increases the risk of TB outbreaks, due to their effects on cellular interactions involved in LTBI [30]. In our findings, Mtb induces TNF-α production in hv-WAD cells, with secretion levels amplified by the virulent H37Rv strain. This confirms the essential role of TNF-α in sustaining LTBI and identifies hv-WAD as a source of this cytokine. We propose that this reflects a LTBI state driven by bacterial metabolic adaptation and continuous antigen secretion, which together facilitate Mtb persistence within this niche. The adipocyte-derived TNF-α may thus contribute to persistent infection as a mechanism of immune evasion.

To further evaluate the significance of our findings, we referred to a previous study that detected the Mtb-specific insertion sequence (IS) 6110 (a repetitive mobile element specific to the Mtb complex, commonly used for TB diagnosis) in autopsies of individuals who died from causes unrelated to TB. This finding was pivotal in understanding LTBI and the potential role of adipose tissue as a reservoir [8]. In our analysis of non-in vitro infected hv-WAD cells from bariatric surgery biopsies, the 42% of patients tested positive for the IS6110 sequence. These positive samples also expressed transcripts for the Ag85 complex, including the 30 kDa and the 5KST sequence. The 30 kDa and 5KST were the most abundant and frequently co-expressed in hv-WAD. Overall, 68% of samples showed expression of at least one Mtb RNA sequence. These data suggest that a panel containing IS6110, the 30 kDa protein, and the 5KST sequence could be effective in detecting latent TB infection in patients undergoing immunosuppressive treatments.

In this study, we describe the invasion of Mtb H37Ra and H37Rv in human visceral adipocytes (hv-WAD) and report the semi-quantitative expression of virulence and dormancy protein transcripts relative to 16S RNA. However, a fully quantitative analysis would require more robust extraction methods to isolate Mtb RNA more efficiently [31,32]. Subsequent studies employing quantitative technologies, such as real-time PCR and RNA sequencing, will be necessary to fully characterize the transcriptomics of Mtb within visceral adipocytes. This includes applying quantitative transcriptomics and functional transcriptomics to identify the metabolic transition of Mtb into a dormant state.

Our findings contribute to growing evidence that questions the granuloma’s function as a protective niche for latent TB. While the transition to a fully dormant state of Mtb was not captured in our model, we have established that hv-WAD cells support a permissive infection and the expression of Mtb genes, including markers linked to dormancy.

## 4. Materials and Methods

### 4.1. Ethics Statement and Patients

The project was reviewed and approved by the Institutional Ethics and Research Committee of the Instituto Nacional de Enfermedades Respiratorias Ismael Cosío Villegas (protocol number: C21-22). All participants were recruited from a hospital in Mexico City and provided written informed consent. Visceral white adipose tissue (WAT) biopsies were obtained from patients undergoing bariatric surgery (gastric bypass or gastric sleeve procedures). Patient identity and all identifiable information were anonymized. General demographic data, reported as ranges, are presented in Table 1.

### 4.2. Human Visceral White Adipocyte Tissue (hv-WAT)

WAT from biopsies of patients were used to isolated adipocytes. These biopsies were processed into single-cell suspensions using C tubes and a gentleMACS™ Tissue Dissoci-ator (Miltenyi Biotec, Auburn, CA, USA). Following dissociation, the tissue was filtered through sterile gauze and centrifuged at 860× *g* for 10 min at 21 °C. The single human visceral white adipocytes (hv-WADs) are localized in the floating layer. The hv-WAD was transferred to medium 199 (M199) (Merck KGaA, Darmstadt, Germany).

### 4.3. Fluorescent Staining of hv-WAD

The single hv-WAD were stained with 5(6)-Carboxyfluorescein diacetate N-succinimidyl ester (CFSE) (Merk KGaA, Darmstadt, Germany; Millipore-Sigma in USA) following standard protocols. Briefly, 1 × 10^6^ hv-WAD were resuspended in 1 mL of staining buffer (PBS containing 0.1% BSA), and 2 µL of CFSE (5 mM) was added. The mixture was incubated at 37 °C with 5% CO_2_ for 10 min. The reaction was stopped by adding five volumes of cold M199, followed by incubation on ice for 5 min. The cells were then centrifuged at 860× *g* for 10 min at 21 °C. After discarding the supernatant, 5 × 10^6^ CFSE-stained hv-WAD were cultured in M199 (Life Technologies, Staley Rd., Grand Island, NY, USA) supplemented with insulin (1.7 µM) (AMSA, Mexico City, Mexico), dexamethasone (1 µM) (Laboratorios PISA, Guadalajara, Mexico), and exosome-free fetal bovine serum 3% (SBI, Palo Alto, CA, USA). The culture was maintained at 37 °C with 5% CO_2_ for 96 h.

### 4.4. Mtb-H37Ra-mCherry and Mtb-H37Rv Strains

*Mycobacterium tuberculosis* H37Ra (Mtb-H37Ra) (ATCC-25177, Rockville, MD, USA) was cultured in Middlebrook 7H9 medium (Becton Dickinson, Sparks, MD, USA) supplemented with albumin-dextrose-catalase (ADC) (Gibco, USA) at 37 °C under constant agitation. For the virulent *Mycobacterium tuberculosis* H37Rv strain (Mtb-H37Rv) (ATCC 25618, Rockville, MD, USA), stable expression of mCherry was achieved using the pCherry8 plasmid (Addgene, Cambridge, MA, USA) [33]. The Mtb-H37Rv strain was cultivated in Middlebrook 7H9 broth medium (Becton Dickinson, Sparks, MD, USA) and stored at −70 °C. Prior to infection, the bacteria were thawed and disaggregated to obtain a single-cell suspension. This was accomplished by vigorous mixing with 3 mm sterile glass beads, followed by centrifugation at 900× *g* for 10 min. The resulting pellet was resuspended in supplemented M199.

### 4.5. Internalization of Mtb-Cherry by hv-WAD

Suspensions of 5 × 10^6^ single-cell hv-WAD alone or with Mtb-H37Ra-Cherry at a multiplicity of infection (MOI) of 20 were incubated at 37 °C with 5% CO_2_ for 48 or 72 h. The multiplicity of infection (MOI) was determined by infecting adipocytes with a range of Mtb concentrations (MOI 1, 5, 20, and 100). MOI 20 was chosen for all subsequent experiments because it achieved the highest infection percentage without affecting adipocyte viability. After incubation, the cells were centrifuged at 2700× *g* for 5 min. The floating cells were transferred to a new tube and centrifuged again at 2700× *g* for 5 min. The supernatant was carefully removed using a syringe, and the hv-WAD-CFSE were added 100 µL of 4% formaldehyde. The fixed cells were then mounted on a microscope slide, covered with a coverslip, and sealed with nail polish. Fluorescence microscopy (AXIO Scope. A1, Zeiss) was used to analyze the cells. To determine the infection percentage, 300–700 cells with a CFSE-positive membrane were counted per field. The percentage of these cells that were also Mtb-Cherry positive was then calculated. To confirm the internalization of Mtb within the hv-WAD, cell nuclei were stained with DAPI (Invitrogen, Carlsbad, CA, USA), and images were captured using a confocal microscope (Olympus FV-1000). For cytokine analysis, the media was filtered through a 0.2 µm pore filter (Corning, NY, USA) to remove non-phagocytosed bacteria and stored at −20 °C until further use.

### 4.6. hv-WAD Infected with Mtb-H37Ra-Cherry or Mtb-H37Rv Strain

Suspensions of 5 × 10^6^ single-cell hv-WAD were cultured in 5 mL tube with 3 mL of M199 (Gibco, Grand Island, NY, USA) supplemented with insulin (1.7 µM) (AMSA, CDMX, Mexico), dexame-thasone (1 µM) (Laboratorios PISA, Guadalajara, Mexico), and exosome-free fetal bovine serum 3% (SBI, Palo Alto, CA, USA) at 37 °C with 5% CO_2_ for 96 h. After incubation, the cells were centrifuged at 2700× *g* for 5 min. The medium was carefully removed using a syringe, and 3 mL of M199 with Mtb-H37Ra or Mtb-H37Rv was added at a multiplicity of infection (MOI) of 20 or 10, respectively. These were incubated at 37 °C with 5% CO_2_ for 48 or 96 h. The media was filtered through a 0.22 µm filter and were stored at −20 °C.

### 4.7. RNA Extraction from hv-WAD

Infected or non-infected hv-WAD were centrifuged at 2700× *g* for 5 min. The floating cells were transferred to a new tube and centrifuged again at 2700× *g* for 5 min to remove the medium, which was carefully aspirated using a syringe. The cell pellet was then lysed added 350 μL of Buffer RLT (QIAGEN, Venlo, The Netherlands) and vortex for 5 min at maximum speed ensured both the hv-WAD and bacteria were lysed. RNA was extracted from each sample using the RNeasy Mini Kit (QIAGEN, Venlo, The Netherlands) following the manufacturer’s protocol. The RNA purity was assessed 1.7–1.9 for hv-WAD; 1.8–1.9 for hv-WAD infected with Mtb-H37Rv, and 1.6–1.7 for hv-WAD infected with Mtb-H37Ra.

### 4.8. Amplification of Mtb-RNA in hv-WAD by PCR

For cDNA synthesis, the SuperScript II RT Kit (Invitrogen, Carlsbad, CA, USA) was used according to the manufacturer’s instructions. The PCR amplification was performed using cDNA as the template and specific forward and reverse primers targeting several Mtb gene fragments. These included virulence-associated genes (19 kDa, 30 kDa, Ag85, ESAT6, CFP10, 5KST, IS6110), dormancy-associated genes (Icl-1, Lip-Y, WhiB3, SodA, Tgs-1), and the housekeeping gene 16s rRNA. The negative control was nuclease-free water, and M. tuberculosis H37Rv genomic DNA was included as a positive control in all reactions (100 pg). The amplified PCR products were separated on an agarose gel (2%), stained with Gel Red (Biotum, Fremont, CA, USA), and visualized under UV light using a ChemiDoc MP imaging system (Bio-Rad, Hercules, CA, USA).

The primers in Table 2 were used to amplify specific sequences of Mtb in lysed hv-WAD [31,32]. 

### 4.9. Densitometric Analysis of Gene Amplifications

Image Lab software version 5.0 (Bio-Rad, Hercules, CA, USA) was used to analyze the gel images. The lane and bands tools were employed to detect the number of bands present in the gels. Relative quantification was performed using the quantify tool to each PCR product. The band intensity and relative quantification values were normalized by 16S RNA.

### 4.10. Immunoassays for IL-1β, IL-8, and TNF-α

The media from non-infected hv-WAD (n = 54), hv-WAD infected with H37Ra (n = 41), or hv-WAD infected with H37Rv (n = 13) were tested for cytokines secretion. The quantitation of TNF-α (Thermo Scientific, Rockford, IL, USA), IL-1β (MabTech, Stockholm, Sweden), and IL-8 (MabTech, Stockholm, Sweden) were determined using a sandwich ELISA. Briefly, 96-well plates were coated with anti-TNF-α (1:200), anti-IL-1β (1:250) or anti-IL-8 (1:250) antibodies and incubated overnight at 4 °C. After discarding the antibody solution, the wells were washed three times with PBS containing 0.05% Tween 80 and blocked with SuperBlock (Thermo Scientific, Rockford, IL, USA) for 30 min (TNF-α) at 37 °C or 1 h (IL-1β and IL-8) at room temperature (RT). Following blocking, the wells were washed five times with wash solution (0.05% Tween 80 in PBS), and the supernatants from the gene expression assays, along with a cytokine standard curve for TNF-α, IL-1β or IL-8, were added. The plates were incubated at 37 °C (TNF-α) or RT (IL-1β and IL-8) for 2 h. After five additional washes, a second biotin-conjugated antibody anti-TNF-α (1:1000), anti-IL-1β (1:500), or anti-IL-8 (1:500) was added, and the plates were incubated for 30 min for TNF-α (Endogend, Paris, France) or 1 h for IL-1β and IL-8 (MabTech, Stockholm, Sweden) at RT. The wells were washed five times, and streptavidin–alkaline phosphatase (Jackson Immunoreseach, West Grove, PA, USA) (1:2000) was added, followed by incubation for 30 min (TNF-α) or 1 h (IL-1β and IL-8) at RT. After five washes, the substrate p-Nitrophenyl Phosphate (Merk KGaA, DA, Germany; Millipore-Sigma, Burlington, MA, USA) was added, and the plates were incubated for 20 min (TNF-α) or 1 h (IL-1β and IL-8) at RT.

### 4.11. Statistical Analysis

Comparisons between groups were performed using the Mann–Whitney test or Wilcoxon signed-rank test in GraphPad Prism version 10.4.0 (Boston, MA, USA), with a significance *p* ≤ 0.05.

## Figures and Tables

**Table 1:** Demographic characteristics of adipose biopsy donors.

	Number	Percentage
Subjects	54	-----
Male	21	38.8
Female	33	61.1
Age (18–65)	41	----
Body Mass Index		
Class II obesity (35.0–39.9)	4	7.4
Class III obesity (>39.9)	40	92.6
Type 2 Diabetes	41	75.9

**Table 2:** Primer for Mtb transcripts.

Target	Primer Sequences 5′	Size (bp)
19 kDa	F: GAGACCACGACCGCGGCAGG R: AATGCCGGTCGCCGCCCCGCCGAT	159
30 kDa (fbpB)	F: TGTACCAGTCGCTGTAGAAG R: GACATCAAGGTTCAGTTC	190
Ag85 (fbpC)	F: AAGGTCCAGTTCCAGGGCG R: ATTGGCCGCCCACGGGCATGAT	154
ESAT6	F: GTCCATTCATTCCCTCCT R: CTATGCGAACATCCCAGT	220
CFP10	F: GGATCCATGGCAGAGATGAAGACC R: GGATCCGAAGCCCATTTGCGAGG	203
5KST	F: TTGCTGAACTTGACCTGCCCGTA R: GCGTCTCTGCCTTCCTCCGAT	128
IS6110	F: CCTGCGAGCGTAGGCGTCGG R: CTCGTCCAGCGCCGCTFCGG	123
rRNA 16S	F: GCCGTAAACGGTGGGTACTA R: TGCATGTCAAACCCAGGTAA	194
Icl-1	F: CGGATCAACAACGCACTGCA R: TTCTGCAGCTCGTAGACGTT	140
LipY	F: GTATTAGCCGCTGCCGAGGA R: GATACCGCTGGCGAATTCACTCT	204
WhiB3	F: TGGACTCATCGATGTTCTTCC R: TAGGGCTCACCGACCTCTAA	142
SodA	F: ACACCTTGCCAGACCTGGA R: CGCCCTTTACGTAGGTGGC	108
Tgs-1	F: AACGAAGACAGTTATTCGAGC R: CTCATACCTTTCATCGGAGAGCC	197

## Data Availability

The original contributions presented in this study are included in the article. Further inquiries can be directed to the corresponding author(s).
